# Influences of hyperlipidemia history on stroke outcome; a retrospective cohort study based on the Kyoto Stroke Registry

**DOI:** 10.1186/s12883-015-0297-1

**Published:** 2015-03-25

**Authors:** Kazuo Shigematsu, Yoshiyuki Watanabe, Hiromi Nakano, on behalf of the Kyoto Stroke Registry Committee

**Affiliations:** Department of Neurology, the National Hospital Organization, Minami Kyoto Hospital, 11 Nakaashihara, Joyo, Kyoto, 610-0113 Japan; Department of Epidemiology for Community Health and Medicine, Kyoto Prefectural University of Medicine, Graduate School of Medical Science, Kyoto, Japan; Department of Neurosurgery, Kyoto Kidugawa Hospital, Kyoto, Japan

**Keywords:** Hyperlipidemia, Stroke, Outcome

## Abstract

**Background:**

Although hyperlipidemia is known as a risk factor of stroke, its effects on the outcome are unknown. The aim of the study is to clarify the influences of hyperlipidemia on the stroke early outcome by estimating odds ratio (OR) for sequelae requiring care and hazard ratio (HR) for death.

**Methods:**

A total of 12617 stroke patients registered in the Kyoto Stroke Registry with information on a hyperlipidemia history. We compared patients who had hyperlipidemia history and patients who hadn’t. The OR for remaining sequelae requiring certain care on 30 day after stroke was calculated using a logistic regression in stroke as a whole and in each stroke subtype; cerebral infarction (CI), cerebral hemorrhage (CH) and subarachnoid hemorrhage (SAH). The HR for death within 30 day after stroke was estimated by the Cox regression.

**Results:**

The OR (95% confidence interval) for remaining sequelae 30 days after stroke was 0.66 (0.60-0.73, p < 0.001) in patients with hyperlipidemia history compared with patients without hyperlipidemia history. After stratified by stroke subtypes, it was 0.75 (0.67-0.85, p < 0.001) in CI, 0.59 (0.45-0.77, p < 0.001) in CH and 0.77 (0.43-1.38, p = 0.767) in SAH. The HR (95% confidence interval) for death was 0.39 (0.31-0.48, p < 0.001) in patients with hyperlipidemia history comparing patients without hyperlipidemia history. After stratified by stroke subtypes, it was 0.45 (0.32-0.63, p < 0.001) in CI, 0.64 (0.44-0.93, p = 0.018) in CH and 0.76 (0.47-1.23, p = 0.264) in SAH. Each value was adjusted for age and sex.

**Conclusions:**

This study suggests that the outcome is favorable for patients with hyperlipidemia history in terms of both remaining sequelae and HR for death. A factor which increases the incidence of the disease could influence on the severity of the disease in a favorable way.

**Electronic supplementary material:**

The online version of this article (doi:10.1186/s12883-015-0297-1) contains supplementary material, which is available to authorized users.

## Background

Elevated levels of cholesterol are reported to be associated with increased rate of recovery from ADL disability [1]. Hyperlipidemia is one of the major risk factors for atherosclerosis [2,3]. A risk factor is regarded as a factor which increases the incidence of a disease [4-9]. Hyperlipidemia is a well-known risk factor for vascular diseases such as stroke and myocardial infarction. Long term expose to the risk factors promotes atherosclerosis and subsequently develops stroke. Complex of factors should be involved in the stroke pathogenesis and the total sum of the risk factors should play a critical role in the development of stroke. Risk factors have been discovered by comparing the incidences of the disease between two groups; a group with a factor and a group without a factor. If the incidence in a group with a factor is higher than in another group without it, the factor is regarded as a risk factor. Severity of the disease is not necessarily taken into consideration in this process. However, when considering stroke, not only the incidence but also the severity are precious, for stroke is important not only as a major cause of death but also as a major cause of sequelae requiring care, which are heavy burden for patients, for caregivers and for the society. To understand the factors which determine the severity of the disease is needed. Whether a risk factor could influence on the severity of stroke is interesting and worthwhile to investigate [10]. To clarify the relationship between a risk factor and a severity of the disease is important because it gives tips to understand the pathogenesis of the disease and leads to the prevention. Besides, among hospitalized disabled older adults, elevated levels of cholesterol are associated with increased rate of recovery from ADL disability [1].

The Kyoto Stroke Registry has a large amount of data of stroke patients with information on hyperlipidemia history and on early outcome; degrees of sequelae and mortality. Based on the registry data, we calculated the odds ratios (OR) for remaining sequelae after 30 days of stroke and the hazard ratios (HR) for death within 30 days. The aim of the study is to investigate the effects of hyperlipidemia history on early outcome of stroke and, by doing so, to answer the question whether a risk factor could affect the outcome of the disease.

## Methods

We reviewed all stroke patients with information on hyperlipidemia history identified in the Kyoto Stroke Registry (KSR) from January 1999 to April 2009. The details of KSR have been previously reported [11-15]. The KSR has been keeping efforts to grasp the state of stroke patients in Kyoto Prefecture aiming at promotion of countermeasures against stroke from the prevention to rehabilitation to improve impairment as much as possible to avoid staying in hospitals and facilities and return to home. Stroke patients are registered in KSR in cooperation with all medical institutions belonging to the Kyoto Medical Association to investigate the actual state of stroke patients in Kyoto Prefecture. The Kyoto Medical Association (KMA) distributes the registration form to all cooperative facilities, and physicians in charge at cooperative facilities fill the form and send it back to the KMA. The registration items include age, sex, date of onset, past medical histories (hypertension, diabetes mellitus (DM), and hyperlipidemia), cigarette smoking and alcohol consumption, blood pressure on consultation, presence or absence of arrhythmia, consciousness level based on the Japan Coma Scale (JCS) [11] and history of examinations (CT, MRI), activity of daily life (ADL) at day 30 after the development, presence or absence of dementia, and survival. Information on the past medical histories was obtained from the patients/their families and from the medical records. Stroke was classified into 3 major subtypes; cerebral infarction (CI), cerebral hemorrhage (CH), and subarachnoid hemorrhage (SAH) according to the WHO definition [16].

We used the following definitions:

1. Systolic and diastolic hypertension: blood pressure is 140 and 90 mm Hg or higher;
2. Diabetes mellitus: fasting plasma glucose is 126 mg/dl or higher, and/or plasma glucose 2 h after 75 g glucose load is 200 mg/dl or higher;
3. Hyperlipidemia: serum cholesterol level is 220 mg/dl or higher and/or triglyceride is 150 mg/dl or higher.
4. Arrhythmia: any types of irregularity of heart beats detected by ECG and/or physical examination.

ADL was evaluated into 4 grades using the ADL scale in Japan:

1. ADL1 (No symptoms or no significant disability), independent gait;
2. ADL2 (Mildly disabled condition), a cane is necessary for gait;
3. ADL3 (Moderately disabled condition), assistance is necessary for gait; and
4. ADL4 (Severely disabled condition), a bedridden state. The date of death was registered when the patient died within 30 days after the event.

Based on information obtained from the patients and their families and on medical records, the patients were classified into 2 groups; those who have hyperlipidemia history (HL) and those who had not (NL).

### Statistical analyses

The frequencies of characteristics in HL and NL were determined and evaluated for univariate associations by χ square analysis. Numerical data such as age and blood pressure were compared with a student-t test. The OR for sequelae requiring certain care was calculated comparing HL with NL using a logistic regression in stroke as a whole and in each stroke subtype, CI, CH and SAH after adjustment for age and sex. The HR for death within 30 days after stroke was estimated comparing HL with NL using the Cox regression. Each value was adjusted for age and sex. The OR was calculated as e^β^ with 95% CI = e^(β±1.96×SE)^. Here, “β” is the regression coefficient corresponding to LD or HD (with AB as a reference), and “SE” is the standard error of “β”. No multiplicity adjustment was applied in principal because a significant difference was detected in the above χ square test and to reduce the error of the second kind, i.e. to reduce the risk of overlooking differences. Analyses were performed using SPSS ver.19 (IBM). All reported p values were 2-sided. Statistical significance was set at p < 0.05.

This research was performed in accordance with the ethical principles for medical research involving human subjects outlined in the Declaration of Helsinki. This research was approved by the Board of Directors, the Kyoto Medical Association, the Department of Health and Welfare, Kyoto Prefecture and the Ethics Committee of the National Hospital Organization, Minami Kyoto Hospital. Since all identifying personal information was stripped from the secondary files before analysis, the boards waived the requirement for written informed consent from the patients involved.

## Results

We reviewed a total of 12617 patients with information on the hyperlipidemia history. Patients’ characteristics are summarized in Table 1. The significance of the difference of each variable between patients with hyperlipidemia and patients without hyperlipidemia are shown in Additional file 1: Table S1. The ORs for consciousness disturbance at the onset of stroke comparing patients with hyperlipidemia to patients without hyperlipidemia calculated using a binominal logistic regression are provided in Additional file 2: Table S2. Most of patients in the study cohort had CT or MRI or both. Neither CT nor MRI was performed in 99 patients; at least one was performed in 99.2% of the patients.

**Table 1:** **Summary of the stroke patients in the study cohort**

	**Stroke (n = 12617)**	**Without hyperlipidemia history (n = 10198)**	**With hyperlipidemia history (n = 2419)**
**Age (mean ± SD)**	71.2 ± 12.9	71.5 ± 13.2	69.8 ± 11.1
**Sex (men % (n))**	55.2 (6962)	55.9 (5696)	52.3 (1266)
**Stroke type (cerebral infarction/cerebral hemorrhage/subarachnoid hemorrhage, % (n))**	67.1/24.8/8.0 (8451/3126/1011)	63.9/27.1/9.0 (6500/2755/918)	80.8/15.4/39 (1951/371/93)
**Consciousness level (JCS0/JCS1/JCS2/JCS3, % (n))**	59.0/19.5/11.5/10.0 (7261/2400/1412/1229)	55.9/20.4/12.4/11.3 (5546/2027/1231/1120)	72.1/15.7/7.6/4.6 (1715/373/181/109)
**Systolic blood pressure (mean ± SD)**	160.9 ± 31.2	160.6 ± 31.5	162.3 ± 29.6
**Diastolic blood pressure (mean ± SD)**	87.5 ± 18.8	87.3 ± 18.8	88.5 ± 18.7
**Hypertension history, with % (n)**	61.8 (7585)	57.5 (5694)	79.7 (1891)
**Diabetes mellitus history, with % (n)**	20.4 (2545)	17.0 (1724)	34.6 (821)
**Cigarette smoking, with % (n)**	32.3 (3440)	31.8 (2702)	34.4 (738)
**Alcohol consumption, with % (n)**	38.1 (3967)	38.2 (3183)	37.5 (784)
**Activity of daily life; ADL1/ADL2/ADL3, % (n)**	50.6/29.1/11.4/8.9 (5363/3086/1212/947)	48.3/29.7/12.0/9.9 (4072/2502/1013/835)	59.1/26.7/9.1/5.1 (1291/584/199/112)
**Mortality, dead % (n)**	8.3 (1047)	9.4 (957)	3.7 (89)

The OR for remaining sequelae after 30 days of stroke (i.e. ADL 2–4 compared with ADL1) using NL as a reference was 0.66 (0.60-0.73, p < 0.001). After stratified by stroke subtypes, it was 0.76 (0.68-0.86) in CI, 0.65 (0.50-0.84, p < 0.001) in CH and 0.83 (0.47-1.45, p = 0.502) in SAH. They are summarized in Table 2.

**Table 2:** **OR for remaining the state requiring certain care in patients with hyperlipidemia compared to patients without hyperlipidemia**

	**Odds ratio**	**95% confidence interval**		**p**
		**Lower**	**Upper**	
Stroke	0.659	0.596	0.729	<0.001
Cerebral infarction	0.764	0.680	0.858	<0.001
Cerebral hemorrhage	0.650	0.503	0.840	0.001
Subarachnoid hemorrhage	0.825	0.469	1.449	0.502

Adjusted for age and sex.

Mortality within 30 days after stroke was lower in HL (3.7%, 89 out of 2411) than in NL (9.4%, 958 out of 10170). The number of missing data on survival information was 8 out of 2419 in HL and 28 out of 10198 in NL, respectively.

**Figure 1:**
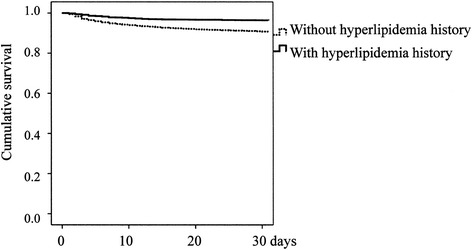
**Kaplan-Meier survival curves of patients with cerebral infarction (p < 0.001).**

**Figure 2:**
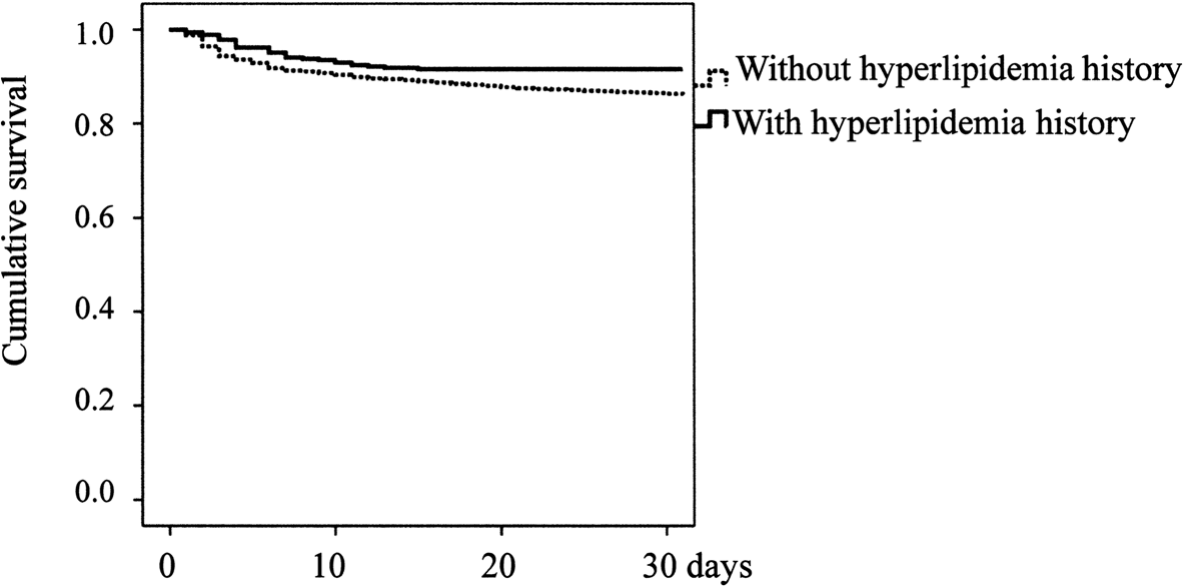
**Kaplan-Meier survival curves of patients with cerebral hemorrhage (p = 0.004).**

**Figure 3:**
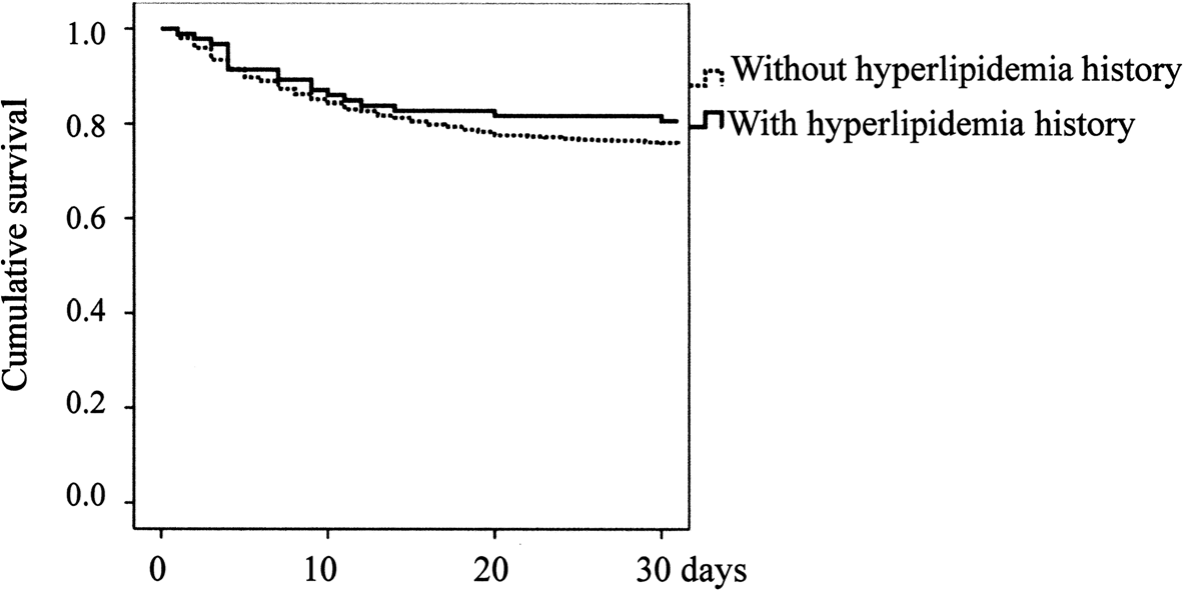
**Kaplan-Meier survival curves of patients with subarachnoid hemorrhage (p = 0.302).**

The Kaplan-Meier Survival curves comparing HL and NL with a log-rank test in CI, in CH and in SAH are presented in Figures 1, 2 and 3, respectively.

The HR for death within 30 days was 0.39 (0.31-0.48, p < 0.001) in stroke. After stratified by stroke subtypes, it was 0.45 (0.32-0.63, p < 0.001) in CI, 0.64 (0.44-0.93, p = 0.018) in CH and 0.76 (0.47-1.23, p = 0.264) in SAH. Each value was adjusted for age and sex. They are summarized in Table 3. Frequency of hyperlipidemia patients with or without medication in the study cohort is shown in Additional file 3: Table S3. The HRs for death within 30 days after stroke in patients with hyperlipidemia compared to patients without hyperlipidemia and compared to with and without medication for hyperlipidemia estimated using the Cox regression are summarized in Additional file 4: Table S4.

**Table 3:** **Hazard ratios for death within 30 days after stroke in patients with hyperlipidemia compared to patients without hyperlipidemia**

	**Hazard ratio**	**95% confidence interval**		**p**
		**Lower**	**Upper**	
Stroke	0.386	0.310	0.480	<0.001
Cerebral infarction	0.450	0.323	0.626	<0.001
Cerebral hemorrhage	0.641	0.444	0.926	0.018
Subarachnoid hemorrhage	0.760	0.470	1.230	0.264

Adjusted for age and sex.

## Discussion

Both the OR for remaining sequelae after stroke and the HR for death were lower in HL than in NL irrespective of age and sex, indicating that hyperlipidemia history had a favorable influence on the early outcome of stroke. Since the early outcome largely depends on the severity of stroke, the results suggest that hyperlipidemia history has a favorable effect on the severity of stroke. As a risk factor, hyperlipidemia history increases the incidence of stroke. Nevertheless, it might make the disease less severe. The great portion of progress of atherosclerosis is asymptomatic. It would be symptomatic only after an obstruction in cerebral flow arises to develop brain functional damage. So, it could be more serious in degree without being symptomatic and the contrary is also the same. Hyperlipidemia accelerates damage on a certain cerebral artery and makes atherosclerosis symptomatic. Possible interpretation, therefore, includes that hyperlipidemia developed stroke at rather early stage of the disease “atherosclerosis”. Namely, hyperlipidemia could develop less serious stroke.

This study showed similar results in CI and CH, not in SAH. Although both CI and CH are the same at the point that they are cerebrovascular disorder based on cerebral arterial deterioration, the pathology of the two is mutually the opposite. The former is the obstruction of the artery and the latter is the rupture of it. There could be differences of the effects of hyperlipidemia on the two distinct pathologies. Iso et al. reported that there was an inverse relation between the serum cholesterol level and the risk of death from hemorrhagic stroke in middle-aged American men [17]. They admitted the positive association of higher serum cholesterol levels with death from nonhemorrhagic stroke and total cardiovascular disease. Elevated levels of cholesterol are reported to be associated with increased rate of recovery from ADL disability [1]. Hyperlipidemia might develop stroke at rather early stage of arteriosclerosis, including less serious stroke.

Major determinants of stroke outcome include age [18], sex [19-21], treatments and severity at the onset. The age and sex were adjusted in the study. The bias due to differences in treatments is unavoidable and we could not adjust for them. However, since treatment according to the guidelines is performed and the governmental medical insurance for the whole nation covers all people in Japan, a treatment bias is not very much likely. Embolism could lead a poor outcome without hyperlipidemia [22,23]. However, influence of embolism should be predominantly on CI. The differences are supposed to be based on the severity of the disease in principle [24]. So, hyperlipidemia history is thought to have an effect on stroke severity. Since the severity of stroke could largely depend on the size and location of the lesion [10,25], effects of hyperlipidemia on cerebral arteries may be different among their sizes and locations. Further studies to clarify the associations between the size and location of the brain lesion and hyperlipidemia are promising and should be feasible at stroke centers with MRI for example.

The study may have all the limitations that a retrospective cohort study could have. The definition of hyperlipidemia in the KSR includes both high level of serum cholesterol and triglyceride, which was a standard in Japan during the period of this survey. Therefore, it is difficult to understand which, cholesterol or triglyceride, accounts for the association. The study with a large cohort together with the previous reports suggests an association between serum lipid and outcome of stroke.

In this study, since analysis was performed using a stroke registry, the subjects consisted of stroke patients. The results were “relative” ones obtained using a certain subgroup (NL in principle) as a reference. For example, a significantly lower OR in HL compared with NL can also be interpreted as a higher OR in NL compared with HL.

## Conclusion

The study showed that the presence/absence of hyperlipidemia history was associated with the outcome of both CI and CH in a favorable manner, irrespective of the age and sex. A risk factor, which increases the incidence of a disease, may influence on the severity of a disease, even inversely and favorably.

## Additional files

Additional file 1: Table S1. The significance of the difference of each variable between patients with hyperlipidemia and patients without hyperlipidemia. Additional file 2: Table S2. Odds ratios for consciousness disturbance at the onset of stroke comparing patients with hyperlipidemia to patients without hyperlipidemia. Additional file 3: Table S3. Frequency of hyperlipidemia patients with or without medication in the study cohort. Additional file 4: Table S4. Hazard ratios for death within 30 days after stroke in patients with hyperlipidemia compared to patients without hyperlipidemia and compared to with and without medication for hyperlipidemia.
