# Supplementary material for: Influences of hyperlipidemia history on stroke outcome; a retrospective cohort study based on the Kyoto Stroke Registry
Source: BMC Neurol. 2015 Mar 25;15:44. doi: 10.1186/s12883-015-0297-1 (PMC4376998; doi:10.1186/s12883-015-0297-1)
Supplement: Additional file 1: Table S1. — The significance of the difference of each variable between patients with hyperlipidemia and patients without hyperlipidemia. [file 12883_2015_297_MOESM1_ESM.docx]

Additional file 1: Table S1. The significance of the difference of each variable between patients with hyperlipidemia and patients without hyperlipidemia

|  | P value |
| --- | --- |
| **Age*** | <0.001 |
| **Sex** | 0.002 |
| **Stroke type (cerebral infarction/cerebral hemorrhage/subarachnoid hemorrhage)** | <0.001 |
| **Consciousness level (JCS0/JCS1/JCS2/JCS3)** | <0.001 |
| **Systolic blood pressure*** | <0.001 |
| **Diastolic blood pressure*** | <0.001 |
| **Hypertension history** | <0.001 |
| **Diabetes mellitus history** | <0.001 |
| **Cigarette smoking** | 0.022 |
| **Alcohol consumption** | 0.518 |
| **Activity of daily life; ADL1/ADL2/ADL3** | <0.001 |
| **Mortality, dead** | <0.001 |

All P values are 2 sided

Chi-square test was used for categorical data

*Student t test was used for numerical data
