# Supplementary material for: Influences of hyperlipidemia history on stroke outcome; a retrospective cohort study based on the Kyoto Stroke Registry
Source: BMC Neurol. 2015 Mar 25;15:44. doi: 10.1186/s12883-015-0297-1 (PMC4376998; doi:10.1186/s12883-015-0297-1)
Supplement: Additional file 2: Table S2. — Odds ratios for consciousness disturbance at the onset of stroke comparing patients with hyperlipidemia to patients without hyperlipidemia. [file 12883_2015_297_MOESM2_ESM.docx]

Additional file 2: Table S2. Odds ratios for consciousness disturbance at the onset of stroke comparing patients with hyperlipidemia to patients without hyperlipidemia

|  | Odds ratio | 95% Confidence Interval | | p |
| --- | --- | --- | --- | --- |
|  |  | Lower | Upper |  |
| Without hyperlipidemia history | Reference | | | |
| Without medication | 0.479 | 0.413 | 0.555 | <0.001 |
| With medication | 0.492 | 0.435 | 0.556 | <0.001 |

Adjusted for age and sex, binominal logistic regression
