# Supplementary material for: Influences of hyperlipidemia history on stroke outcome; a retrospective cohort study based on the Kyoto Stroke Registry
Source: BMC Neurol. 2015 Mar 25;15:44. doi: 10.1186/s12883-015-0297-1 (PMC4376998; doi:10.1186/s12883-015-0297-1)
Supplement: Additional file 3: Table S3. — Frequency of hyperlipidemia patients with or without medication in the study cohort. [file 12883_2015_297_MOESM3_ESM.docx]

Additional file 3: Table S3. Frequency of hyperlipidemia patients with or without medication in the study cohort

|  | Medication for hyperlipidemia | Frequency | Percent |
| --- | --- | --- | --- |
| With hyperlipidemia history | Not applicable | 10198 | 80.8 |
| Without hyperlipidemia history | Without medication | 1000 | 7.9 |
|  | Under medication | 1419 | 11.2 |
