# Supplementary material for: Influences of hyperlipidemia history on stroke outcome; a retrospective cohort study based on the Kyoto Stroke Registry
Source: BMC Neurol. 2015 Mar 25;15:44. doi: 10.1186/s12883-015-0297-1 (PMC4376998; doi:10.1186/s12883-015-0297-1)
Supplement: Additional file 4: Table S4. — Hazard ratios for death within 30 days after stroke in patients with hyperlipidemia compared to patients without hyperlipidemia and compared to with and without medication for hyperlipidemia. [file 12883_2015_297_MOESM4_ESM.docx]

Additional file 4: Table S4. Hazard ratios for death within 30 days after stroke in patients with hyperlipidemia compared to patients without hyperlipidemia and compared to with and without medication for hyperlipidemia

|  | Hazard Ratio | 95% Confidence Interval | | P |
| --- | --- | --- | --- | --- |
|  |  | Lower | Upper |  |
| Without hyperlipidemia history^*1^ | Reference | | | |
| Without medication^*1^ | 0.320 | 0.224 | 0.458 | <0.001 |
| With medication^*1^ | 0.424 | 0.325 | 0.553 | <0.001 |
| Without hyperlipidemia history^*2^ | Reference | | | |
| Without medication^*2^ | 0.335 | 0.234 | 0.479 | <0.001 |
| With medication^*2^ | 0.420 | 0.322 | 0.547 | <0.001 |

^*1^Crude

^*2^Adjusted for age and sex
